# Inanspruchnahme der Influenza-Impfung bei Personen über 60 Jahren: Räumliche Unterschiede und regionale Einflussfaktoren

**DOI:** 10.1007/s00103-025-04103-8

**Published:** 2025-08-18

**Authors:** Manas K. Akmatov, Claudia Kohring, Jakob Holstiege, Doreen Müller

**Affiliations:** Fachbereich Epidemiologie und Versorgungsatlas, Zentralinstitut für die kassenärztliche Versorgung, Salzufer 8, 10587 Berlin, Deutschland

**Keywords:** Räumliche Cluster, Räumliche Regression, Räumliche Variationen, Regionale Risikofaktoren, Influenza-Impfung, Senioren, Spatial clusters, Spatial regression, Spatial variation, Regional risk factors, Influenza vaccination, Seniors

## Abstract

**Hintergrund:**

Die Ziele der Studie sind die Berechnung der Influenza-Impfquote bei Personen ab 60 Jahren, die Untersuchung räumlicher Variationen und die Identifizierung räumlicher Cluster sowie der regionalen Risikofaktoren für eine niedrige Impfquote.

**Methoden:**

Als Datengrundlage dienten die bundesweiten vertragsärztlichen Abrechnungsdaten der Jahre 2022 und 2023. Die Studienpopulation bildeten 22.303.411 gesetzlich Versicherte ab 60 Jahren. Berechnet wurde die Influenza-Impfquote als Anteil geimpfter Versicherter in der Saison 2022/2023 an allen Versicherten mit Vertragsarztkontakt im Jahr 2022. Untersucht wurden die globale räumliche Autokorrelation, lokale Cluster sowie regionale Risikofaktoren für eine niedrige Impfquote mittels *Global* und *Local Moran’s I* sowie geografisch gewichteter Regression.

**Ergebnisse:**

Die bundesweite Influenza-Impfquote betrug 37 %, auf Kreisebene variierte sie zwischen 10–61 %. Die globale Autokorrelation lag bei 0,87 (*p* < 0,0001). Lokal zeigten sich 2 stark ausgedehnte Cluster. Ein großes Cluster mit relativ hohen Impfquoten erstreckte sich über alle östlichen Bundesländer und Kreise im Südosten Niedersachsens, Schleswig-Holstein sowie Hamburg. Ein weiteres Cluster mit relativ niedrigen Impfquoten umfasste fast alle Kreise in Süddeutschland. Die Risikofaktoren für eine niedrige Impfquote waren Wohnkreis in Westdeutschland sowie in dünn besiedelten ländlichen Kreisen. Die Hausarztdichte wies einen positiven Zusammenhang mit der Impfquote auf. Die Zusammenhänge zwischen der Impfquote und den erklärenden Variablen zeigten regionale Variationen.

**Diskussion:**

Die Identifizierung räumlicher Cluster sowie von Risikofaktoren für eine niedrige Impfquote kann als Grundlage für regional zugeschnittene Interventionsmaßnahmen zur Verbesserung der Impfinanspruchnahme dienen.

**Zusatzmaterial online:**

Zusätzliche Informationen sind in der Online-Version dieses Artikels (10.1007/s00103-025-04103-8) enthalten.

## Abstract (en)

**Background:**

The aims of the study were to determine the influenza vaccination coverage among individuals aged 60 and older, examine spatial variations, and identify spatial clusters as well as regional risk factors for low vaccination coverage.

**Methods:**

The study was based on nationwide outpatient claims data from 2022 and 2023. The study population consisted of 22,303,411 individuals with statutory health insurance aged 60 and older. The influenza vaccination coverage was calculated as the proportion of vaccinated individuals during the 2022/23 season among all persons with Statutory Health Insurance (SHI) who had contact with SHI-authorized physicians in 2022. Global spatial autocorrelation, local spatial clusters, and regional risk factors for low vaccination coverage were examined using global and local Moran’s I as well as geographically weighted regression.

**Results:**

The nationwide influenza vaccination coverage was 37%, with district-level variations ranging from 10% to 61%. The global autocorrelation was 0.87 (global Moran’s I, *p* < 0.0001). Two strongly pronounced local clusters were identified. A large spatial cluster with relatively high vaccination coverage spanned across all eastern federal states as well as districts in southeastern Lower Saxony, Schleswig-Holstein, and Hamburg. Another cluster with relatively low vaccination coverage encompassed almost all districts in southern Germany. The risk factors for low vaccination coverage included residence in western Germany and in sparsely populated rural districts. The density of general practitioners showed a positive correlation with vaccination coverage. The relationships between vaccination coverage and explanatory variables varied regionally.

**Discussion:**

The identification of spatial clusters and regional risk factors for low vaccination coverage can serve as a basis for regionally tailored intervention measures to improve vaccine uptake.

## Hintergrund

Es ist unbestritten, dass Impfungen zu den wirksamsten Präventionsmaßnahmen gegen viele Infektionskrankheiten gehören [1]. Aus primärpräventiver Sicht sind Impfungen insbesondere bei bestimmten Bevölkerungsgruppen von immenser Bedeutung, wie z. B. Menschen mit chronischen Grunderkrankungen oder älteren Personen, da diese deutlich anfälliger gegenüber Infektionskrankheiten sind und ein höheres Risiko aufweisen, schwerwiegende Folgen nach Infektionskrankheiten zu entwickeln [2]. Diese Folgen umfassen längere Krankheitsverläufe, häufigere Hospitalisierungen und eine erhöhte Mortalität [3]. Dies stellt nicht nur auf individueller Ebene eine große Belastung dar, sondern führt zu einer erhöhten Beanspruchung des Gesundheitssystems, was Ressourcenknappheit und höhere Gesamtkosten mit sich bringen kann. Impfungen tragen neben dem direkten Schutz der Geimpften zudem zur Reduktion der Krankheitslast in der Gesamtbevölkerung bei, indem sie die Verbreitung von Erregern eindämmen und damit vulnerable Gruppen auch indirekt schützen [4, 5].

Aus diesen Gründen empfiehlt die Ständige Impfkommission (STIKO) aktuell für Menschen im Alter von über 60 Jahren 4 Impfungen: gegen COVID-19, Influenza, Pneumokokken und Herpes Zoster [6]. Seit August 2024 empfiehlt die STIKO außerdem eine einmalige Impfung gegen respiratorische Synzytial-Viren (RSV) für Menschen ab einem Alter von 75 Jahren sowie Menschen im Alter von 60 bis 74 Jahren mit einer schweren Grunderkrankung [6]. Die Impfquoten sind jedoch in dieser Bevölkerungsgruppe auf einem niedrigen Niveau in Deutschland [7]. So ließen sich lediglich 38 % der Menschen ab 60 Jahren in der Saison 2023/2024 gegen Influenza impfen [7]. Noch geringer fiel die Impfquote für Pneumokokken im Jahr 2020 aus; lediglich 20 % der Menschen in Deutschland zwischen 60 und 69 Jahren hatten sich für die Impfung gegen Pneumokokken entschieden [7].

Die individuelle Impfinanspruchnahme ist das Ergebnis eines komplexen Entscheidungsprozesses und wird von vielen verschiedenen Faktoren beeinflusst [8]. Faktoren auf der Individualebene, wie z. B. persönliche Einstellungen gegenüber Impfungen, impf- oder erkrankungsspezifisches Wissen, Risikowahrnehmung [9], sowie verschiedene soziodemografische Faktoren wie sozioökonomischer Status spielen eine wichtige Rolle, wobei der sozioökonomische Status sowohl mit höheren als auch mit niedrigeren Impfquoten einhergehen kann und durch Wissen, Einstellungen und Risikowahrnehmung mediiert wird [10, 11]. Aber auch spezifische regionale Faktoren sind relevant – so kann es beispielsweise räumliche Cluster mit hohen oder niedrigen Impfquoten geben [12]. Dies zeigen Studienerkenntnisse zu regionalen Unterschieden in der Impfinanspruchnahme. Beobachtet wurden Unterschiede in verschiedenen geografischen Regionen in Deutschland. Die Impfquoten sind beispielsweise für einige Impfungen in den östlichen Bundesländern höher als in den westlichen [13]. Zwischen den Bundesländern bestehen ebenfalls deutliche Unterschiede [7]. Auch kleinräumige Variationen in den Impfquoten wurden beobachtet, z. B. für die Kinderimpfungen gegen Masern [14] oder humane Papillomviren (HPV; [15]) oder für Impfungen im Erwachsenenalter (Impfung gegen saisonale Influenza bei Schwangeren [16] oder bei Menschen mit chronischen Erkrankungen [17]). Erklärungen hierfür können regional unterschiedliche Versorgungsstrukturen sein, wie z. B. unterschiedliche Erreichbarkeit von impfenden Ärzt*innen. Aber auch regional wirksame gesundheitspolitische Maßnahmen könnten die Impfbereitschaft beeinflussen [18]. Regionale Faktoren der Impfinanspruchnahme auf Basis von bundesweiten Daten wurden bisher nicht untersucht. Es finden sich lediglich vereinzelte, regional begrenzte Studien, wie z. B. die Untersuchung der Inanspruchnahme der Masern-Mumps-Röteln-Impfung in Westfalen-Lippe [19].

Basierend auf einer Vollerfassung der Daten aller gesetzlich Krankenversicherten in Deutschland verfolgte die vorliegende Studie folgende Ziele: a) die Berechnung der Influenza-Impfquote bei Personen ab 60 Jahren, b) die Untersuchung räumlicher Variationen und die Identifizierung räumlicher Cluster am Beispiel der Influenza-Impfung und c) die Identifizierung von regionalen Risikofaktoren für eine niedrige Impfquote.

## Methoden

### Datengrundlage und Studienpopulation

Als Datengrundlage dienten die bundesweiten vertragsärztlichen Abrechnungsdaten für alle Versicherten der Gesetzlichen Krankenversicherung (GKV) in Deutschland, die mindestens einmal in den Jahren 2022 und 2023 eine vertragsärztliche Leistung in Anspruch genommen haben. Diese Daten werden gemäß § 295 Fünftes Sozialgesetzbuch (SGB V) erfasst. Die Abrechnungsdaten werden von den 17 Kassenärztlichen Vereinigungen (KV) bereitgestellt. Der Datenkörper umfasst alle von den Vertragsärzt*innen dokumentierten Diagnosen sowie alle abgerechneten kollektivvertraglichen Leistungen, zu denen auch Impfungen gehören, sofern sie aufgrund einer STIKO-Empfehlung in die Schutzimpfungs-Richtlinie und damit in den GKV-Leistungskatalog aufgenommen wurden. Der Datenkörper enthält keine Leistungen, die im Rahmen der Hausarztzentrierten Versorgung (HzV) erbracht wurden. Dies ist vor allem in Baden-Württemberg, Bayern und Westfallen-Lippe der Fall. Zusätzlich stehen Angaben zu Geschlecht, Alter und Wohnkreis der Versicherten zur Verfügung. Die Studienpopulation bildeten weibliche und männliche Versicherte ab 60 Jahren (*N* = 22.303.411).

### Influenza-Impfung

Die STIKO empfiehlt die saisonale Influenza-Impfung für Menschen ab 60 Jahren, Personen, die einer Risikopopulation angehören oder solche Risikopersonen betreuen, sowie Schwangere [6]. Die ersten Impfungen gegen Influenza innerhalb einer Saison werden in der Regel im September durchgeführt. Die vertragsärztlichen Daten stehen auf Quartalsebene zur Verfügung. In der vorliegenden Studie werden Influenza-Impfungen berücksichtigt, die zwischen dem dritten Quartal des Jahres 2022 und dem ersten Quartal des Jahres 2023 abgerechnet wurden (d. h. Saison 2022/2023, Abrechnungen zwischen Juli 2022 und März 2023; [20]). Die in diesen 3 Quartalen durchgeführten Impfungen gegen die saisonale Influenza wurden anhand der KV-spezifischen Abrechnungspositionen identifiziert, die weitgehend den Dokumentationsziffern der Schutzimpfungs-Richtlinie des Gemeinsamen Bundesausschusses (G-BA) entsprechen [21]. Die im zweiten Quartal des Jahres 2023 abgerechneten Impfungen werden in dieser Analyse nicht berücksichtigt, da die vorangegangene Influenzasaison in den Monaten April bis Juni weitgehend abgeschlossen war. Berechnet wurde die Impfquote als Anteil der Versicherten in der Altersgruppe ab 60 Jahren, bei dem in den 3 genannten Quartalen eine Influenza-Impfung abgerechnet wurde, an allen Versicherten dieser Altersgruppe im Jahr 2022. Aufgrund der abweichenden Kriterien für die Zusammensetzung der Studienpopulation wurde die Influenza-Impfquote entsprechend der für diese Studie spezifizierten Charakteristika berechnet und weist leichte Abweichungen von der vom Robert Koch-Institut (RKI) berichteten Impfquote auf [7].

### Statistische Auswertung

#### Untersuchung globaler Autokorrelation und Identifizierung lokaler räumlicher Cluster.

Das Vorhandensein einer räumlichen Autokorrelation bei der Inanspruchnahme von Influenza-Impfungen in der Altersgruppe ab 60 Jahren (d. h. Impfquote) wurde mittels *Global Moran’s I* mit einer reihenstandardisierten Raumgewichtungsmatrix untersucht [22]. Für die Erstellung der räumlichen Gewichtungsmatrix wurde die Queen-Contiguity-Methode verwendet. Diese Methode wird meist als Default-Methode für polygonale Daten empfohlen, die unterschiedliche Flächengrößen aufweisen [23]. Dies ist der Fall in Deutschland, wo die Kreisfläche stark zwischen ca. 36 km^2^ (Stadt Schweinfurt) und 5470 km^2^ (Mecklenburgische Seenplatte) variiert. Die Werte für *Global Moran’s I* können zwischen −1 und 1 liegen und werden wie andere Korrelationskoeffizienten interpretiert: Ein Wert nahe 1 weist auf eine starke positive räumliche Autokorrelation hin, was bedeutet, dass sich benachbarte Regionen hinsichtlich der untersuchten Variable ähneln, während ein Wert nahe −1 zeigt, dass sich benachbarte Regionen eher unähnlich sind. Ein Wert um 0 signalisiert das Fehlen einer Autokorrelation und lässt zufällige Verteilungen über den Raum annehmen.

Als nächster Schritt wurde das *Local Moran’s I* (*Local Indicators of Spatial Association*, LISA) angewandt, um lokale räumliche Cluster zu identifizieren [24]. Dabei können die einzelnen Regionen in 4 verschiedene Clustertypen eingeteilt werden:

- Regionen mit hohen Impfquoten, die von Regionen mit ähnlich hohen Impfquoten umgeben sind (Clustertyp „hoch-hoch“),
- Regionen mit niedrigen Impfquoten, die an Regionen mit ähnlich niedrigen Impfquoten angrenzen (Clustertyp „niedrig-niedrig“),
- Regionen mit einer hohen Impfquote, die von Regionen mit niedrigen Impfquoten umgeben sind (Clustertyp „hoch-niedrig“) oder
- Regionen mit einer niedrigen Impfquote, die an Regionen mit einer hohen Impfquote angrenzen (Clustertyp „niedrig-hoch“).

Aus der räumlichen Auftretenshäufigkeit der Regionen mit bestimmten Typzugehörigkeiten können anschließend Rückschlüsse auf Cluster gezogen werden. Die räumliche Autokorrelation sowie die Untersuchung lokaler räumlicher Cluster wurden mit dem R‑Paket „spdep“, Version 1.2–8 [25], durchgeführt.

#### Untersuchung regionaler Indikatoren.

Die abhängige Variable für die regressionsanalytische Untersuchung war die Inanspruchnahme von Influenza-Impfungen auf Kreisebene (d. h. Influenza-Impfquote). Folgende erklärende Variablen aus dem INKAR-Datensatz (Indikatoren und Karten zur Raum- und Stadtentwicklung, [26]) wurden untersucht: Anzahl der Hausärzt*innen pro 10.000 Einwohner*innen (d. h. Hausarztdichte), wohnungsnahe hausärztliche Grundversorgung in Metern (d. h. Entfernung zum(r) nächsten Hausarzt*in), Anteil der ausländischen Bevölkerung an den Einwohner*innen in Prozent, Anteil der Schulabgänger*innen ohne Schulabschluss an den Schulabgänger*innen in Prozent, durchschnittliches Haushaltseinkommen in Euro je Einwohner*in und Anteil der Arbeitslosen an den zivilen Erwerbspersonen in Prozent. Zusätzlich wurden 2 kategoriale Variablen als Dummy-Variablen ins Modell aufgenommen: a) Ost-(einschließlich Berlin)-West-Kreise sowie b) 4 siedlungsstrukturelle Kreistypen gemäß Bundesinstitut für Bau‑, Stadt- und Raumforschung ([27]; kreisfreie Großstädte, städtische Kreise, ländliche Kreise mit Verdichtungsansätzen und dünn besiedelte ländliche Kreise). Die Auswahl der zu untersuchenden erklärenden Variablen basierte auf theoretischen Überlegungen sowie bisher bekanntem Wissen über Risikofaktoren der unzureichenden Impfinanspruchnahme [13, 28, 29]. Die genaue Beschreibung der erklärenden Variablen sowie deren geografische Verteilung sind im Onlinematerial zu sehen (Tabelle A1 und Abbildung A1). Die Untersuchungseinheit aller eingeschlossenen Variablen bildeten die 401 Kreise gemäß der administrativen Struktur zum 31.12.2016. Die regionale Verteilung dieser Variablen ist im Onlinematerial abrufbar. Die Variablen aus dem INKAR-Datensatz stammen aus den Jahren 2021 und 2022. Da anzunehmen ist, dass kreisstrukturelle Faktoren sich zeitlich langfristiger entwickeln und beständiger sind als die jährliche Impfquote, werden die in dieser Arbeit untersuchten Kreisindikatoren trotz des ökologischen Designs als regionale Risikofaktoren bezeichnet.

Zunächst wurden alle definierten Risikofaktoren auf Kreisebene sowohl univariabel als auch multivariabel mittels globaler linearer Regression auf ihren Zusammenhang mit der Influenza-Impfquote untersucht. Alle Variablen, die im multivariablen Modell statistisch signifikant mit der Impfquote assoziiert waren, wurden im nächsten Schritt auf Multikollinearität mittels *Variance Inflation Factor* (VIF) überprüft. Anschließend wurden sie in die geografisch gewichtete Regressionsanalyse (GWR) aufgenommen [30]. Die GWR ist eine Art lokale Version der globalen linearen Regression und ermöglicht, für jedes räumliche Gebiet eine eigene lokal angepasste Regressionsgleichung zu erstellen. Die Gewichtung der benachbarten Kreise erfolgt durch eine adaptive Kernel-Funktion, die die Entfernung zwischen den Kreisen und die Anzahl der Kreise berücksichtigt. Näher gelegene benachbarte Kreise werden im Modell stärker gewichtet als weiter entfernte Kreise. Die GWR-Analyse wurde mit dem R‑Paket „spgwr“, Version 06–37 [31], durchgeführt. Das Signifikanzniveau wurde auf 5 % festgelegt, aufgrund des explorativen Vorgehens erfolgte keine Korrektur für multiples Testen [32]. Zudem gehen die klassischen Korrekturverfahren wie Bonferroni von unabhängigen Tests aus, welche bei räumlichen Daten mit benachbarten Regionen meist nicht gegeben sind. Die statistische Auswertung erfolgte mit R, Version 4.3.1.

## Ergebnisse

### Impfquote, räumliche Autokorrelation und Cluster

**Abb. 1:**
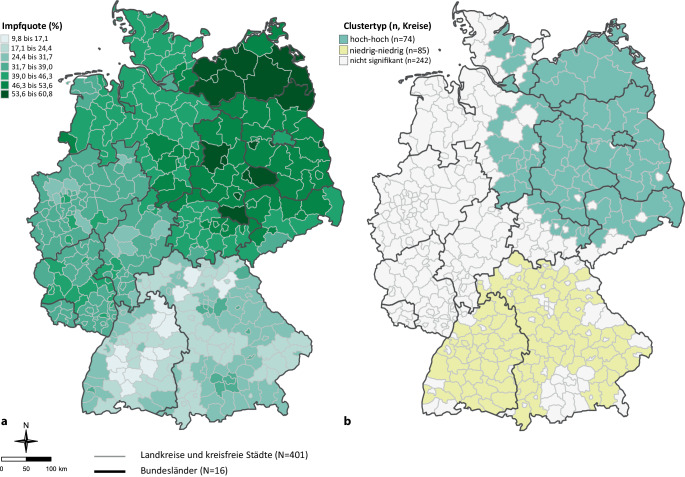
Kreisspezifische Influenza-Impfquoten (**a**) und räumliche Cluster mit hohen und niedrigen Influenza-Impfquoten (**b**)^a^. (^a^ Räumliche Cluster des Typs „hoch-hoch“ (*grün*) und „niedrig-niedrig“ (*gelb*) wurden anhand eines LISA-Modells (Local Moran’s I) identifiziert [24]. 401 Kreise gemäß administrativer Struktur zum 31.12.2016). *LISA* Local Indicators of Spatial Association

Die bundesweite Influenza-Impfquote unter den GKV-Versicherten ab 60 Jahren in der Saison 2022/2023 lag bei 37 %. Die Impfquote war höher in den östlichen Bundesländern (50 %) sowie in Berlin (46 %) im Vergleich zu den westlichen Bundesländern (33 %). Auf Kreisebene variierte die Impfquote um den Faktor 6 zwischen 10 % im Kreis Schwäbisch Hall (Baden-Württemberg) und 61 % in Magdeburg (Sachsen-Anhalt; Abb. 1a).

Die globale Autokorrelation lag bei 0,87 (*Global Moran’s I, p* < 0,0001), was darauf hindeutet, dass sich benachbarte Regionen hinsichtlich ihrer Impfquoten tendenziell ähneln. Lokal zeigten sich 2 stark ausgedehnte Cluster des Typs „hoch-hoch“ und „niedrig-niedrig“. Ein großes Cluster mit relativ hohen Impfquoten erstreckte sich über die östlichen Bundesländer mit allen Kreisen in Mecklenburg-Vorpommern und nahezu allen Kreisen in Sachsen-Anhalt und Brandenburg sowie Berlin. Zusätzlich gehörten zu diesem Cluster Kreise im Südosten Niedersachsens, in Schleswig-Holstein sowie in Hamburg (Abb. 1b). Der Clustertyp „niedrig-niedrig“ umfasste die meisten Kreise im Süden Deutschlands.

### Globale und lokale Risikofaktoren für eine niedrige Influenza-Impfquote

Im globalen multivariablen linearen Modell waren 6 Variablen statistisch signifikant mit der Impfquote assoziiert (Tab. 1). Die VIF-Werte für alle 6 Variablen lagen unter 3,50 und deuten damit auf leichte, aber unproblematische Multikollinearität hin (Tab. 1). Den stärksten Zusammenhang mit der Impfquote zeigte die Variable „Ost-West“; in den östlichen Kreisen war die Influenza-Impfquote um knapp 10 % höher als in den westlichen Kreisen. Weiterhin war die Impfquote um 3 % niedriger in dünn besiedelten ländlichen Kreisen im Vergleich zu kreisfreien Großstädten. Eine höhere Hausarztdichte war mit einer höheren Influenza-Impfquote assoziiert, jede zusätzliche Hausärztin bzw. jeder zusätzliche Hausarzt erhöhte die Impfquote um 1,2 %. Ein positiver Zusammenhang war auch für Arbeitslosenquote zu verzeichnen; mit einem Anstieg der Arbeitslosenquote um 1 % stieg die Impfquote um 2,57 %. Ein höherer Anteil ausländischer Personen hing mit geringeren Impfquoten zusammen; hier sank die Impfquote um 0,72 % mit einem Anstieg des Ausländeranteils um 1 %. Das Modell mit allen untersuchten Variablen erklärte 52 % der regionalen Unterschiede bei den Impfquoten.

**Tab. 1:** Zusammenhänge zwischen Influenza-Impfquoten und erklärenden Variablen auf Kreisebene – Ergebnisse der globalen linearen Regression sowie der lokalen geografisch gewichteten Regression (GWR)

Variablen	Mittelwert (SD) oder *n*	Univariable lineare Regression		Multivariable lineare Regression^a^			Multivariable geografisch gewichtete Regression^b^		
		Regressionskoeffizient	*P*-Wert	Regressionskoeffizient	*P*-Wert	VIF	1. Quintil	Median	3. Quintil
Anzahl der Hausärzt*innen je 10.000 Einwohner*innen	6,2 (0,6)	3,648	< 0,0001	1,234	0,032	1,10	0,691	1,870	2,770
Ausländeranteil an den Einwohner*innen, %	12,9 (5,6)	−0,698	< 0,0001	−0,725	< 0,0001	2,34	−0,331	−0,117	0,019
Anteil der Schulabgänger*innen ohne Abschluss, %	7,1 (2,5)	1,792	< 0,0001	−0,053	0,759	1,61	–	–	–
Haushaltseinkommen in € je Einwohner*innen	2012 (194)	−0,024	< 0,0001	0,006	0,021	2,06	0,002	0,007	0,012
Arbeitslosenquote, %	5,2 (2,2)	2,445	< 0,0001	2,575	< 0,0001	2,49	0,174	1,396	2,594
Entfernung zum(r) nächsten Hausarzt*in, Meter	1155 (583)	0,001	0,168	–	–	–	–	–	–
*Region*^*c*^									
Östliche Kreise, einschließlich Berlin, *n*	77	17,077	< 0,0001	9,815	< 0,0001	1,83	5,141	9,196	14,673
Westliche Kreise, *n*	324	Referenz	–	Referenz	–	–	Referenz	Referenz	Referenz
*Kreistyp*^*c*^									
Dünn besiedelte ländliche Kreise, *n*	100	−0,203	0,902	−3,010	0,036	3,41	−2,740	−1,206	1,249
Ländliche Kreise mit Verdichtungsansätzen, *n*	101	−1,557	0,345	−1,948	0,159	3,19	–	–	–
Städtische Kreise, *n*	134	−4,835	0,002	−1,298	0,302	3,10	–	–	–
Kreisfreie Großstädte, *n*	66	Referenz	–	Referenz	–	–	Referenz	Referenz	Referenz
Adjustiertes R‑Quadrat (R^2^)	–	–	–	0,52 (0,60^d^)	–	–	–	0,91	–
Akaike’s Information Criterion (AIC)	–	–	–	2690(2686^d^)	–	–	–	2155	–

Beispielinterpretation (multivariable lineare Regression): Jeder zusätzliche Hausarzt bzw. jede zusätzliche Hausärztin ist mit einer 1,2 % höheren Impfquote assoziiert. Mit einer Zunahme des Ausländeranteils um 1 % sinkt die Impfquote um 0,7 %. Das Modell erklärt 52 % der regionalen Variation in der Influenza-Impfquote auf Kreisebene (R-Quadrat)

Beispielinterpretation (multivariable geografisch gewichtete Regression): Die Stärke der Zusammenhänge zwischen der Impfquote und den erklärenden Variablen weist regionale Variationen auf. Beispielsweise liegt der Regressionskoeffizient für Anzahl der Hausärzt*innen zwischen 0,691 (1. Quintil) und 2,770 (3. Quintil) mit Median von 1,870 je nach Kreis. Mit anderen Worten: Jeder zusätzliche Hausarzt bzw. jede zusätzliche Hausärztin ist in manchen Kreisen mit 0,69 % höherer Impfquote assoziiert, während in anderen Kreisen dieser Wert deutlich höher ist (2,77 %)

^a^ Verallgemeinertes lineares Modell adjustiert für alle Variablen in der Tabelle

^b^ Geografisch gewichtetes Modell adjustiert für die Variablen, die im verallgemeinerten linearen Modell statistisch signifikant assoziiert waren, d. h. Hausärztezahl, Ausländeranteil, Haushaltseinkommen, Arbeitslosenquote, Region und Kreistyp (dünn besiedelte ländliche Kreise vs. kreisfreie Großstädte)

^c^ Die kategorialen Variablen wurden in das Modell als Dummy-Variablen eingeschlossen

^d^ Der AIC-Wert in den Klammern steht für das Modell mit den Variablen, die im geografisch gewichteten Modell untersucht wurden, um die Vergleichbarkeit der AIC-Werte zu gewährleisten

*€* Euro, *n* absolute Anzahl, *SD* Standardabweichung, *VIF* Variance Inflation Factor

**Abb. 2:**
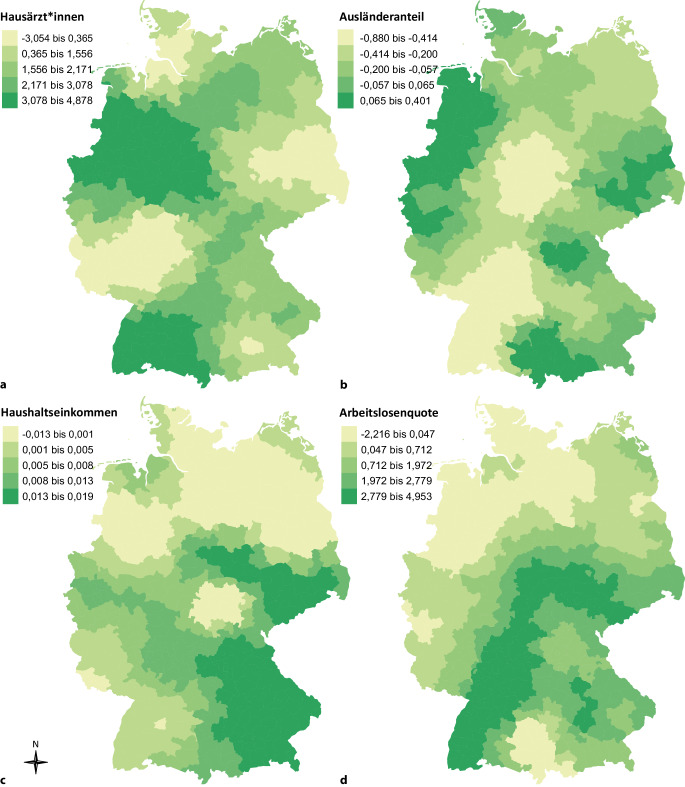
Kreisspezifische Regressionskoeffizienten der erklärenden Variablen im Zusammenhang mit den Influenza-Impfquoten – Ergebnisse der multivariablen geografisch gewichteten Regressionsanalyse (GWR)^a^. **a** Hausärzt*innen, **b** Ausländeranteil, **c** Haushaltseinkommen, **d** Arbeitslosenquote. (^a^ Geografisch gewichtetes Modell adjustiert für alle Variablen in der Abbildung sowie Region (Ost vs. West) und siedlungsstruktureller Kreistyp (dünn besiedelte ländliche Kreise vs. kreisfreie Großstädte; Tab. 1). 401 Kreise gemäß administrativer Struktur zum 31.12.2016)

Die Stärke der Zusammenhänge zwischen der Impfquote und den erklärenden Variablen wies regionale Unterschiede auf (Tab. 1 und Abb. 2). Zudem zeigte das lokale GWR-Modell eine bessere Modellgüte (Tab. 1, AIC-Werte). Außerdem erhöhte sich die durch die untersuchten Variablen erklärte Varianz auf 91 %. Der positive Zusammenhang zwischen der Impfquote und der Hausarztdichte war im Süden von Baden-Württemberg, im Südwesten von Niedersachsen, im Norden von Hessen und in Westfalen-Lippe am stärksten ausgeprägt. Der Anteil der ausländischen Bevölkerung hatte die stärksten negativen Zusammenhänge mit der Impfquote im Nordwesten von Baden-Württemberg und in Teilen von Nordhessen, Südniedersachsen und im Nordwesten von Thüringen. Positive Zusammenhänge mit dem Anteil der ausländischen Bevölkerung wurden ebenfalls beobachtet, wie z. B. im Westen von Niedersachsen oder in Teilen von Nordrhein-Westfalen (Abb. 2).

## Diskussion

Diese Studie hatte neben der Ermittlung der Influenza-Impfquote zum Ziel, räumliche Muster in der Inanspruchnahme von Impfungen zu analysieren und potenzielle Einflussfaktoren zu identifizieren. Dabei wurde insbesondere untersucht, inwiefern soziodemografische und strukturelle Merkmale mit der Impfquote in Zusammenhang stehen. Die hier errechnete Impfquote von 37 % für Personen ab 60 Jahren entspricht den in der Literatur berichteten Werten [7], was die Validität unserer Ergebnisse bestätigt und die methodische Robustheit unterstreicht.

Die Analyse der räumlichen Cluster zeigt, dass sich innerhalb Deutschlands benachbarte Regionen hinsichtlich ihrer Impfquote tendenziell ähneln. Hierbei gibt es 2 ausgeprägte räumliche Cluster. Zum einen zeigte sich ein „Hoch-hoch“-Cluster in Ostdeutschland, aber auch in benachbarten westlichen Kreisen (z. B. Ostniedersachsen und im östlichen Teil von Schleswig-Holstein). Die höhere Influenza-Impfquote in Ostdeutschland im Vergleich zu Westdeutschland ist kein neuer Befund und wurde bereits bei einigen anderen Impfungen sowohl bei Kindern und Jugendlichen (z. B. gegen HPV [15]) als auch bei Erwachsenen (z. B. gegen Pneumokokken [33] oder Diphtherie-Tetanus-Pertussis [13]) beobachtet. Ein Teil der Ost-West-Unterschiede bei der Influenza-Impfquote wurde durch die untersuchten Variablen erklärt. Während die Impfquote in der univariablen Analyse in Ostdeutschland um 17 Prozentpunkte höher war als in Westdeutschland, verringerte sich der Unterschied auf knapp 10 Prozentpunkte nach der Kontrolle für weitere Variablen, der Unterschied blieb jedoch statistisch signifikant. Die im Vergleich zu Westdeutschland etwas höhere Impfinanspruchnahme in Ostdeutschland könnte auf eine höhere Impfakzeptanz in der Bevölkerung zurückzuführen sein. In der ehemaligen DDR hatten präventivmedizinische Maßnahmen eine hohe Relevanz [34], was die auch heute noch vergleichsweise hohen Influenza-Impfquoten der älteren Bevölkerung begünstigen könnte.

Zum anderen fand sich ein „Niedrig-niedrig“-Cluster in Süddeutschland, was durch eine stärkere Impfzurückhaltung beeinflusst sein könnte, die in einigen Bevölkerungsgruppen verbreitet ist. Manche Studien zeigen, dass gerade in wohlhabenderen Regionen mit höherem durchschnittlichen Bildungsniveau teilweise eine höhere Impfskepsis besteht [11]. Es ist jedoch nicht auszuschließen, dass bestimmte regionale Besonderheiten in der ambulanten Versorgung zum Unterschied der Impfquoten beitragen können. Das Vorhandensein von Selektivverträgen, die vor allem in Baden-Württemberg und Bayern verbreitet sind, könnte beispielsweise Teile der regionalen Unterschiede erklären (siehe Abschnitt „Stärken und Limitationen“).

Für die genauere Betrachtung dieser regionalen Unterschiede sind Analysen zu regionalen Risikofaktoren für eine niedrige Impfquote hilfreich. So spielen vermutlich auch strukturelle Barrieren, wie die Verfügbarkeit von impfenden Ärzt*innen oder Impfstoffen, die Entfernung zur nächsten Impfstelle und verfügbare Impfangebote, eine Rolle [18]. Unsere Studie zeigt, dass in ländlichen Regionen mit einer geringeren Hausarztdichte die Inanspruchnahme von Impfungen niedriger ausfällt, während in urbanen Gebieten mit gut ausgebauten Gesundheitsangeboten die Impfquoten höher sind. Bis zu 90 % aller Impfungen in Deutschland werden von niedergelassenen Ärzt*innen durchgeführt. Der überwiegende Anteil davon findet wiederum in Hausarztpraxen statt. In einer früheren Untersuchung haben wir gezeigt, dass etwa 90 % aller Influenza-Impfungen von Hausärzt*innen durchgeführt werden [35]. Wenig überraschend ist daher der Befund zum positiven Zusammenhang zwischen der Impfquote und der Hausarztdichte.

Neben diesen strukturellen Faktoren spielen regional variierende soziodemografische Faktoren, wie beispielsweise die demografische Zusammensetzung der Bevölkerung und daraus resultierende Barrieren (z. B. Sprachbarrieren), ebenfalls eine wichtige Rolle [18]. Insgesamt 3 soziodemografische Variablen zeigten in unserer Analyse einen signifikanten Zusammenhang mit der Impfquote: Ausländeranteil, Arbeitslosenquote und Haushaltseinkommen. Ein möglicher Erklärungsansatz für den negativen Zusammenhang zwischen dem Ausländeranteil und der Impfquote kann möglicherweise durch Sprachbarrieren in dieser Bevölkerungsgruppe erklärt werden, die vor allem bei älteren Menschen mit Migrationsgeschichte vorliegen [36, 37]. In anderen Studien zeigen Personen mit Migrationshintergrund eine geringere Inanspruchnahme niedergelassener Ärzt*innen insgesamt, was sich ebenfalls in den niedrigeren Impfquoten widerspiegeln könnte [38]. Der positive Zusammenhang zwischen der regionalen Arbeitslosen- und Impfquote ist zunächst nicht eindeutig zu interpretieren. Möglich wäre es, dass in Regionen mit höherer Arbeitslosenquote auch die Krankheitslast insgesamt erhöht ist, was eine häufigere Inanspruchnahme ambulanter Versorgungsstrukturen mit sich bringen könnte. Dieser erhöhte Kontakt zu Ärzt*innen könnte zur Folge haben, dass mehr Menschen mit Impfangeboten konfrontiert sind und sich daher auch impfen lassen. Weiterhin können auch soziale Normen und Netzwerke einen Einfluss auf das Impfverhalten haben. In Regionen mit einer hohen Akzeptanz von Impfungen steigt die Wahrscheinlichkeit, dass sich Einzelpersonen impfen lassen, da Impfungen als soziale Norm wahrgenommen werden. Umgekehrt können in Regionen mit einer stärkeren impfkritischen Haltung, verstärkt durch soziale Medien oder lokale Gruppendynamiken, niedrigere Impfquoten beobachtet werden [39]. Leider stehen Daten zur Akzeptanz von Impfungen auf regionaler Ebene nicht zur Verfügung.

### Stärken und Limitationen

Es handelt sich um eine Vollerfassung aller GKV-Versicherten mit vertragsärztlicher Leistungsinanspruchnahme in Deutschland, die knapp 90 % der Wohnbevölkerung ausmacht. Aus diesem Grund gehen wir von einer hohen Repräsentativität der Studienpopulation aus. Zu den Limitationen der Studie gehört eine mögliche Unterschätzung der Impfquote in einigen Regionen Deutschlands, in denen Selektivverträge im Rahmen der HzV angeboten werden. Die dort erbrachten Leistungen werden nicht über das KV-System abgerechnet und sind somit in unserem Datenkörper nicht enthalten. Bundesweit nehmen knapp 10 Mio. Personen an der HzV teil. Regionale Daten zur Anzahl der Personen, die im Rahmen der HzV versorgt werden, existieren nicht. Im Onlinematerial stellen wir eine Übersicht über die Anzahl der Kassen mit bestehenden HzV-Verträgen nach 17 KV-Bereichen (Abbildung A2).

Manche berufstätige GKV-Versicherte über 60 Jahren werden im Rahmen einer arbeitsmedizinischen Maßnahme betriebsärztlich gegen Influenza geimpft. Diese Impfungen fehlen ebenfalls in unserem Datenkörper. Der Datenkörper enthält keine Daten zu Privatversicherten, die etwa 11 % der deutschen Bevölkerung ausmachen. Die Impfinanspruchnahme in dieser Bevölkerungsgruppe ist weitgehend unbekannt.

Eine weitere Limitation der Studie sind der ökologische Ansatz und ein daraus möglicherweise resultierender ökologischer Fehlschluss der Untersuchung regionaler Risikofaktoren für eine niedrige Impfquote. Die dargestellten Assoziationen basieren auf einer Analyse von auf Kreisebene aggregierten Daten und können somit nicht als ursächliche Zusammenhänge auf individueller Ebene interpretiert werden. Vielmehr handelt es sich um eine explorativ-deskriptive Analyse. In bestimmten Fällen (z. B. bei hoher Multikollinearität der erklärenden Variablen) kann die GWR-Analyse zu Scheinkorrelationen führen. Ebenfalls besteht die Gefahr einer Überanpassung des Modells beim Einschluss von mehreren erklärenden Variablen. Weiterhin können sich die Ergebnisse je nach gewählter räumlicher Aggregationsebene (z. B. Kreise, Gemeinde, Postleitzahl) unterscheiden. Die kleinräumigeren Analysen der Impfinanspruchnahme bieten detailliertere Ergebnisse, sind jedoch aufgrund der aktuellen Datenschutzbestimmungen auf Bundesebene nicht möglich.

## Fazit

Die Ergebnisse der vorliegenden Studie liefern wichtige Hinweise darauf, dass regionale Faktoren die Impfakzeptanz und -beteiligung beeinflussen können. Die Identifizierung räumlicher Cluster sowie von Risikofaktoren für eine niedrige Impfquote kann als zusätzliche Grundlage für regional zugeschnittene Interventionsmaßnahmen zur Verbesserung der Impfinanspruchnahme dienen. Aus den Befunden lassen sich gezielte Empfehlungen ableiten, um Impfstrategien in bestimmten Bevölkerungsgruppen oder Regionen zu optimieren. Bemerkenswert ist die Tatsache, dass die untersuchten Variablen 91 % der regionalen Unterschiede bei der Influenza-Impfquote erklärt haben.

## Supplementary Information

Tabelle A1. Beschreibung der ausgewählten Indikatoren; Tabelle A2. Regressionskoeffizienten mit 95 % Konfidenzintervallen und *P*-Werten; Abbildung A1. Verteilung der erklärenden Variablen auf Kreisebene; Abbildung A2. Anzahl der Krankenkassen mit HzV-Verträgen nach KV-Bereichen

## Data Availability

Die während der vorliegenden Studie analysierten Datensätze zur Bestimmung der Influenza-Impfquote auf Individualebene sind aufgrund der aktuellen Datenschutzbestimmungen des Sozialgesetzbuches (SGB V) nicht öffentlich zugänglich. Die untersuchten soziodemografischen Indikatoren aus dem INKAR-Datensatz wurden unter www.inkar.de veröffentlicht.
