# Supplementary material for: Utilization of the influenza vaccination among individuals over 60 years: spatial variations and regional risk factors
Source: Bundesgesundheitsblatt Gesundheitsforschung Gesundheitsschutz. 2025 Aug 18;68(10):1138–46. [Article in German] doi: 10.1007/s00103-025-04103-8 (PMC12460560; doi:10.1007/s00103-025-04103-8)
Supplement: Supplementary file 1 — Tabelle A1. Beschreibung der ausgewählten Indikatoren; Tabelle A2. Regressionskoeffizienten mit 95 % Konfidenzintervallen und P-Werten; Abbildung A1. Verteilung der erklärenden Variablen auf Kreisebene; Abbildung A2. Anzahl der Krankenkassen mit HzV-Verträgen nach KV-Bereichen [file 103_2025_4103_MOESM1_ESM.docx]

**Onlinematerial**

**Tabelle A1.** Beschreibung der ausgewählten Indikatoren

| **Indikatoren** | **Beschreibung** | **Bezugsjahr** |
| --- | --- | --- |
| Hausärzt*innen^a^ | hausärztlich tätige Allgemeinmediziner*innen, inkl. Angestellte je 10.000 Einwohner*innen | 2022 |
| Ausländeranteil^a^ | Anteil der Ausländer*innen an den Einwohner*innen in % | 2022 |
| Schulabgänger*innen ohne Abschluss^a^ | Anteil der Schulabgänger*innen ohne Hauptschulabschluss an den Schulabgänger*innen in % | 2022 |
| Haushaltseinkommen^a^ | durchschnittliches Haushaltseinkommen in € je Einwohner*in | 2021 |
| Arbeitslosenquote^a^ | Anteil der Arbeitslosen an den zivilen Erwerbspersonen in % | 2021 |
| Wohnungsnahe Grundversorgung Hausarzt/Hausärztin^a^ | Einwohnergewichtete Luftliniendistanz zum nächsten Hausarzt bzw. zur nächsten Hausärztin in Meter | 2021 |
| Siedlungsstruktureller Kreistyp mit 4 Ausprägungen^b^: | | 2016 |
| 1) dünn besiedelte ländliche Kreise (100 Kreise) | Kreise mit einem Bevölkerungsanteil in Groß- und Mittelstädten unter 50 % und Einwohnerdichte ohne Groß- und Mittelstädte unter 100 Einwohner*innen pro km² |  |
| 2) ländliche Kreise mit Verdichtungsansätzen (101 Kreise) | Kreise mit einem Bevölkerungsanteil in Groß- und Mittelstädten von mindestens 50 %, aber einer Einwohnerdichte unter 150 Einwohner*innen pro km², sowie Kreise mit einem Bevölkerungsanteil in Groß- und Mittelstädten unter 50 % mit einer Einwohnerdichte ohne Groß- und Mittelstädte von mindestens 100 Einwohner*innen pro km² |  |
| 3) städtische Kreise (134 Kreise) | Kreise mit einem Bevölkerungsanteil in Groß- und Mittelstädten von mindestens 50 % und einer Einwohnerdichte von mindestens 150 Einwohner*nnen pro km^2^; sowie Kreise mit einer Einwohnerdichte ohne Groß- und Mittelstädte von mindestens 150 Einwohner*innen pro km^2^ |  |
| 4) kreisfreie Großstädte (66 Kreise) | kreisfreie Städte mit mindestens 100.000 Einwohner*innen |  |
| ^a^ Bundesinstitut für Bau-, Stadt- und Raumforschung (BBSR). INKAR - Indikatoren und Karten zur Raum- und Stadtentwicklung. <https://www.inkar.de> ^b^ Bundesinstitut für Bau-, Stadt- und Raumforschung (BBSR). Siedlungsstruktureller Kreistyp. <https://www.bbsr.bund.de/BBSR/DE/forschung/raumbeobachtung/Raumabgrenzungen/deutschland/kreise/siedlungsstrukturelle-kreistypen/kreistypen.html> 401 Kreise gemäß der administrativen Struktur zum 31.12.2016 | |  |

**Tabelle A2.** Regressionskoeffiziente mit 95 % Konfidenzintervallen und P-Werten

| **Variablen** | **Univariable lineare Regression** | | | | **Multivariable lineare Regression^a^** | | | |
| --- | --- | --- | --- | --- | --- | --- | --- | --- |
|  | **Regressions-koeffizient** | **Untere 95 % Konfidenz-intervall** | **Obere 95 % Konfidenz-intervall** | **P-Wert** | **Regressions-koeffizient** | **Untere 95 % Konfidenz-intervall** | **Obere 95 % Konfidenz-intervall** | **P-Wert** |
| Anzahl der Hausärzt*innen je 10.000 Einwohner*innen | 3,648 | 1,979 | 5,317 | <0,0001 | 1,234 | 0,104 | 2,365 | 0,032 |
| Anteil der Ausländer*innen an den Einwohner*innen, % | -0,698 | -0,870 | -0,526 | <0,0001 | -0,725 | -0,904 | -0,546 | <0,0001 |
| Anteil der Schulabgänger*innen ohne Abschluss, % | 1,792 | 1,409 | 2,175 | <0,0001 | -0,053 | -0,390 | 0,285 | 0,759 |
| Haushaltseinkommen, € je Einwohner*in | -0,024 | -0,028 | -0,019 | <0,0001 | 0,006 | 0,001 | 0,011 | 0,021 |
| Arbeitslosenquote, % | 2,445 | 2,028 | 2,862 | <0,0001 | 2,575 | 2,096 | 3,055 | <0,0001 |
| Entfernung zum(r) nächsten Hausarzt*in, Meter | 0,001 | -0,001 | 0,03 | 0,168 | - | - | - | - |
| Region^b^ |  |  |  |  |  |  |  |  |
| östliche Kreise, einschließlich Berlin, n | 17,077 | 15,024 | 19,130 | <0,0001 | 9,815 | 7,547 | 12,082 | <0,0001 |
| westliche Kreise, n | Referenz |  |  |  | Referenz |  |  |  |
| Kreistyp^b^ |  |  |  |  |  |  |  |  |
| dünn besiedelte ländliche Kreise, n | -0,203 | -3,441 | 3,035 | 0,902 | -3,010 | -5,827 | -0,193 | 0,036 |
| ländliche Kreise mit Verdichtungsansätzen, n | -1,557 | -4,789 | 1,675 | 0,345 | -1,948 | -4,663 | 0,766 | 0,159 |
| städtische Kreise, n | -4,835 | -7,906 | -1,764 | 0,002 | -1,298 | -3,761 | 1,165 | 0,302 |
| kreisfreie Großstädte, n | Referenz |  |  |  | Referenz |  |  |  |
| Beispielinterpretation (multivariable lineare Regression): Jeder zusätzliche Hausarzt bzw. jede zusätzliche Hausärztin ist mit einer 1,2 % höheren Impfquote assoziiert. Mit einer Zunahme des Ausländeranteils um 1 % sinkt die Impfquote um 0,7 %. Das Modell erklärt 52 % der regionalen Variation in der Influenza-Impfquote auf Kreisebene (R-Quadrat).  ^a^ Verallgemeinertes lineares Modell adjustiert für alle Variablen in der Tabelle.  ^b^ Die kategorialen Variablen wurden in das Modell als Dummy-Variablen eingeschlossen.  €, Euro; n, absolute Anzahl | | | | | | | | |

**Abbildung A1**. Verteilung der erklärenden Variablen auf Kreisebene


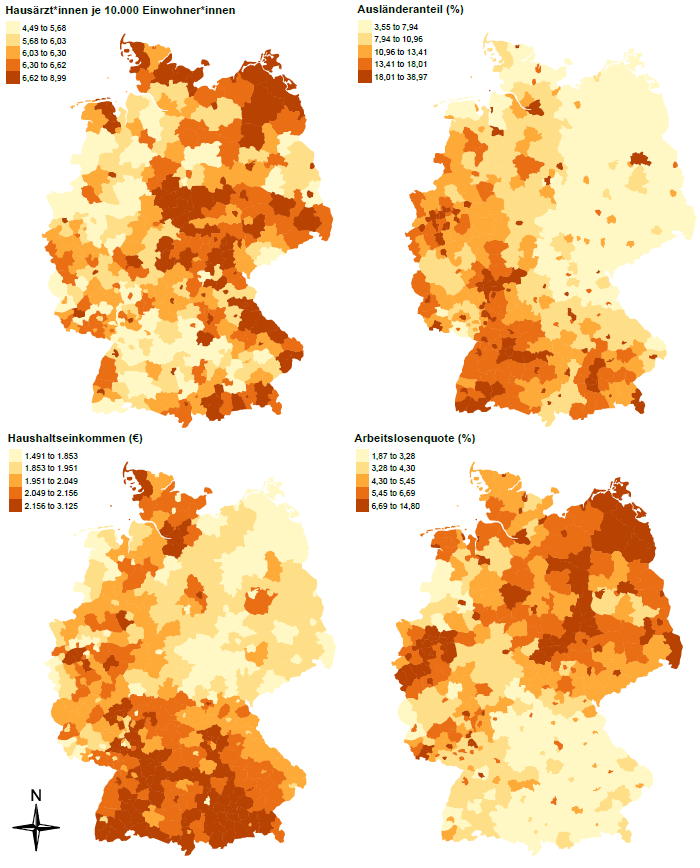


Datenquelle: Bundesinstitut für Bau-, Stadt- und Raumforschung (BBSR). INKAR - Indikatoren und Karten zur Raum- und Stadtentwicklung. <https://www.inkar.de> (eigene Darstellung)

**Abbildung A2.** Anzahl der Krankenkassen mit HzV-Verträgen nach KV-Bereichen


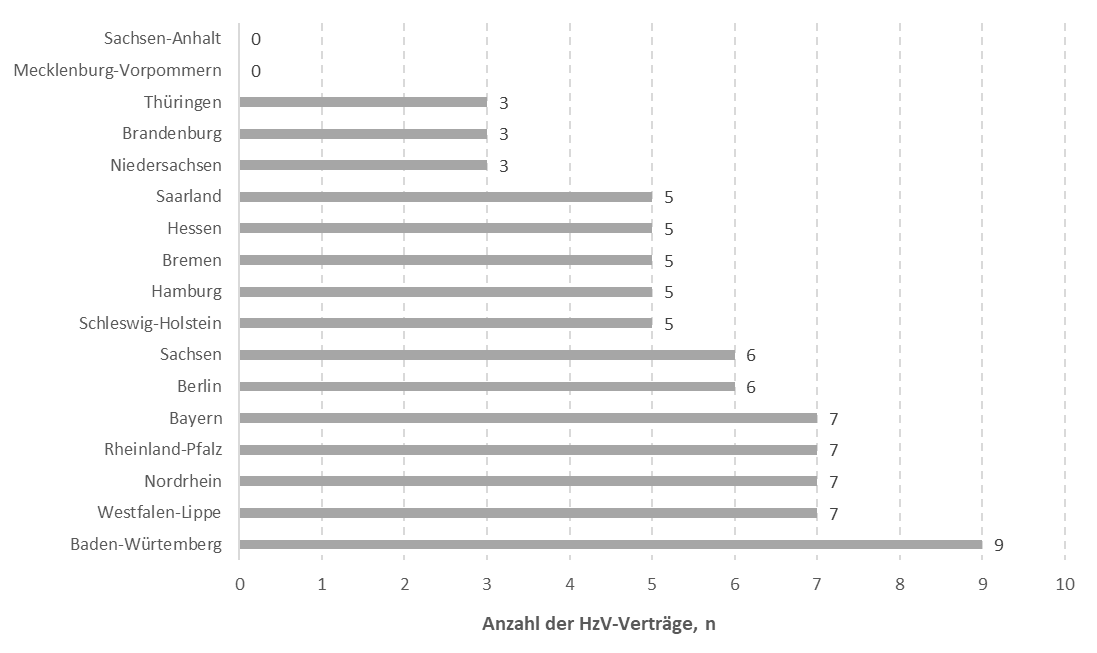


Quelle: <https://www.haev.de/hausarztvertraege/hzv-vertraege-schnellsuche> (Stand: 01.06.2025, eigene Darstellung). HzV = Hausarztzentrierte Versorgung, KV = Kassenärztliche Vereinigung
